# 

**Published:** 1882-03

**Authors:** 


					﻿.Vol. TL
MARCH; 1882.
''No. 3.'
JPHE
pOMCEOPATHlC PHY^ICIAJI,
A MONTHLY JOURNAL OF MEDICAL SCIENCE.
“ Mayna est, veritas, et prc^valebit.''
IE. CT. LEE, 2LZL ID.
EDITOR.
CONTENTS:
(See inside of Coyer.)
PHILADELPHIA:
No. 2109 CHLESTISnjT STREET.
X. 01ST x> o 3ST.
ALFRED HEATH & CO., Some Agents eor Great Britain",
114 Ebury Street, Eaton Square.
Yearly Subscription. $2.00, invariably in advance.
Entered at the Philadelphia Post-Office, as Second-Class Mail Matter,
				

